# Expression profiles of m6A RNA methylation regulators, PD-L1 and immune infiltrates in gastric cancer

**DOI:** 10.3389/fonc.2022.970367

**Published:** 2022-08-08

**Authors:** Zhiyuan Xu, Qiuli Chen, Lilu Shu, Chunye Zhang, Wenjun Liu, Peter Wang

**Affiliations:** ^1^ Department of Gastric Surgery, the Cancer Hospital of the University of Chinese Academy of Sciences (Zhejiang Cancer Hospital), Institutes of Basic Medicine and Cancer (IBMC), Chinese Academy of Sciences, Hangzhou, China; ^2^ Key Laboratory of Prevention, Diagnosis and Therapy of Upper Gastrointestinal Cancer of Zhejiang Province, Hangzhou, China; ^3^ Department of Research and Development, Zhejiang Zhongwei Medical Research Center, Hangzhou, Zhejiang, China; ^4^ National University of Singapore (Suzhou) Research Institute, Suzhou, China

**Keywords:** m6A, PD1, PDL1, TME, time, gastric cancer

## Abstract

Gastric cancer is the fourth most frequent cancer and has a high death rate. Immunotherapy represented by PD-1 has brought hope for the treatment of advanced gastric cancer. Methylation of the m6A genes is linked to the onset and progression of numerous cancers, but there are few studies on gastric cancer. The main purpose of this study aims to analyze the relationship between m6A RNA methylation regulators, PD-L1, prognosis and tumor immune microenvironment (TIME) in gastric cancer. The Cancer Genome Atlas (TCGA) and Genotype Tissue Expression (GTEx) databases were used to acquire transcriptomic data and clinical information from gastric cancer patients. The changes in m6A regulator expression levels in gastric cancer tissues and normal tissues were studied. Consensus clustering analysis was used to separate gastric cancer samples into two categories. We employed Least Absolute Shrinkage, Selection Operator (LASSO) Cox regression analysis, Gene Set Enrichment Analysis (GSEA), and cBioPortal to analyze the m6A regulators, PD-L1 and TIME in gastric cancer. In gastric cancer tissues, the majority of m6A regulatory factors are considerably overexpressed. Two gastric cancer subgroups (Cluster1/2) based on consensus clustering of 21 m6A regulators. PD-L1 and PD-1 expression levels were significantly higher in gastric cancer tissues, and they were significantly linked with METTL3, WTAP, HNRNPD, ZC3H7B, METTL14, FTO, PCIF1, HNRNPC, YTHDF1 and YTDHF2. Cluster1 showed a large increase in resting memory CD4^+^ T cells, regulatory T cells, naïve B cells, active NK cells, and resting Mast cells. Cluster1 and Cluster2 were shown to be involved in numerous critical signaling pathways, including base excision repair, cell cycle, nucleotide excision repair, RNA degradation, and spliceosome pathways. Gastric cancer RiskScores based on prognostic factors have been found as independent prognostic indicators. The amount of tumor-infiltrating immune cells is dynamically affected by changes in the copy number of m6A methylation regulators associated with TIME.

## Introduction

Gastric cancer is a major health crisis because it is the fourth most common cancer worldwide and has the second-highest death rate compared with other cancers (1). Countries in East Asia, South America and Eastern Europe have a high death rate from stomach cancer (2). According to Cancer Statistics, there will be 26,380 new cases of gastric cancer in 2022. The estimated death toll is 6,690 for men and 4,400 for women (3). The treatments for gastric cancer include endoscopy, surgery (laparoscopic surgery without laparotomy), chemotherapy, and radiation (4). Immunotherapy is a novel treatment following surgery, chemotherapy and radiotherapy (5, 6). It achieves a therapeutic effect by inducing, enhancing or regulating immune response. Immunotherapy for gastric cancer currently consists primarily of immune checkpoint inhibitor therapy (7, 8).

Programmed cell death protein 1 (PD-1) is normally expressed on activated immune cells, where it binds to programmed death-ligand 1 (PD-L1) to generate co-inhibitory signals (9, 10). Available data suggest that PD-L1 can be detected in the pathological tissues of about 65% of gastric cancer patients, and its interaction with PD-1 is beneficial for evading immune surveillance (11). Therefore, blocking the combination of the PD-1 and PD-L1 is beneficial for anti-tumor effects. Several studies have shown that PD-L1 is hardly expressed in normal gastric tissue, while its expression level is significantly up-regulated in gastric cancer tissue (12, 13). In China, PD-L1 was found to be highly expressed in nearly half of stage II and III gastric cancer patients, and its high expression indicated a poor prognosis (14). The results of keynote-012, a clinical trial examining the efficacy of pembrolizumab in patients with PD-L1 positive advanced gastric cancer, were reported (15). The results showed that 53% of patients experienced tumor regression and 22% achieved a partial radiographic response, with a median duration of 40 weeks (15). At the same time, pembrolizumab was more toxic than standard second-line chemotherapy. The authors believe that this study brings hope for the treatment of advanced gastric cancer, and there are still many unclear questions in the field of immunotherapy for advanced gastric cancer, which need further investigations (15).

The gastric cancer tumor microenvironment (TME) is a complex and comprehensive system with the characteristics of hypoxia, acidosis, interstitial hypertension and immune-inflammatory response, which affects the proliferation, invasion and metastasis of gastric cancer (16). Monitoring and regulating the TME of gastric cancer are effective means for cancer prevention and targeted therapy. At present, there is still a lack of understanding of the microenvironment of gastric cancer, and it is still impossible to accurately reflect the changes of the entire microenvironment in the body (17). Recently, the researchers found that the expression of N6-methyladenosine (m6A) is closely related to tumor mutation, as well as tumor-infiltrating neutrophil recruitment (18). This has important implications for the impact of anti-tumor immune responses in the TME and for postoperative survival assessment and chemotherapy response prediction in patients with gastric cancer (19). Therefore, the main purpose of this paper aims to analyze the relationship between m6A RNA methylation regulators, PD-1/PD-L1, prognosis and TIME in gastric cancer.

## Methods

### Data collection

Included data were obtained from The Cancer Genome Atlas (TCGA). The data included mRNA expression data and corresponding clinicopathological data of 437 gastric cancer patients, including survival status, survival time, age, gender, grade, and TNM stage. We further obtained the expression profiles and clinical details of these identified genes from the TCGA STAD cohort.

### Selection of m6A regulators

Twenty-four genes (ZC3H13, RBM27, YTHDF1, YTHDF2, YTHDC1, YTHDC2, RBM15, ZC3H7B, YTHDF3, PCIF1, TRA2A, ZCCHC4, HNRNPC, METTL14, WTAP, FTO, ALKBH5, GNL3, METTL3, HNRNPD, YWHAG, CAPRIN1, MSI2, VIRMA) were considered canonical m6A regulators. The expression of 21 m6A regulators was extracted from mRNA expression data. We have used Gene Ontology (GO) to gain a preliminary understanding of the biological functions of 24 m6A methylation regulators. Mutation was analyzed using the maftools package.

### Establishment of a prognostic model

LASSO regression analysis was performed to construct a risk profile model associated with m6A regulators. The formula for calculating the RiskScore is as follows:


$$\text{risk score}=\sum_{i=1}^{n}coef*xi$$

(coef means coefficient; xi means transformed expression value)

Kaplan-Meier analysis was used to analyze differences in OS between high-risk and low-risk groups. The area under the curve was calculated using the receiver operating characteristic (ROC) curve to examine the RiskScore’s predictive potential (AUC). The R package “heatmap” with heatmaps was used to depict the distribution of clinicopathological traits in high and low risk categories. Cox regression models were employed in univariate and multivariate studies to see if the RiskScore was an independent prognostic factor when paired with other clinical factors.

### Estimation of TME cell infiltration

R language was used to estimate the proportion of TME immune matrix components in different clusters, and the results were presented as ImmuneScore, StromalScore and EstimateScore. The cells involved include 22 types of immune cells, such as T regulatory cells, activated NK cells, CD8 T cells, naïve B cells, etc.

### Cell culture and transfection

The human gastric cancer cell lines, HGC-27 and BGC-823, were cultured in RPMI-1640 medium supplemented with 1% penicillin-streptomycin and 10% fetal bovine serum. The cells were maintained in a humidified incubator with 5% CO_2_ at 37°C. Human METTL3 cDNAs were subcloned into pLenti-C-mGFP vector. The pLenti-C-mGFP vector was used as an empty vector control. Specific small hairpin RNAs (shRNAs) targeting METTL3 (shMETTL3) and the control shRNA (shNC) were obtained (GenePharma, Shanghai, China). Lipofectamine 2000 was used for transfection as described before (20).

### Western blotting analysis

The transfected cells were lysed with RIPA buffer supplemented with protease inhibitor cocktail. The supernatant of lysates was collected and measured by a bicinchoninic acid (BCA) assay for protein qualification. Proteins were loaded onto SDS-PAGE and then transferred onto a PVDF membrane. After membrane was blocked with 5% non-fat milk, each primary antibody, including METTL3 (1:1000), PD-L1 (1:1,000) and Tubulin (1:1,000), was added for overnight at cold room. The membranes were incubated with the secondary antibody for 1 h at room temperature. Then, enhanced chemiluminescence approach was conducted to measure the protein signals (21).

### Statistical analysis

The data were presented as mean standard deviation (SD). To examine differences between two and more groups, the student t-test and one-way analysis of variance (ANOVA) were used, respectively. The Kaplan-Meier method and the Log-rank test were used to create survival curves. There is a significant difference between the two groups when p-value is less than 0.05. To assess independent predictive markers, researchers used multivariate Cox regression. The forest plot R software was used to visualize the results. ROC and AUC curves were used to analyze the specificity and sensitivity of m6Ascores using the ROC of R programme. R software was used to run all statistical tests (version 3.6.1).

## Results

### Expression difference of m6A regulators in gastric cancer

**Figure 1:**
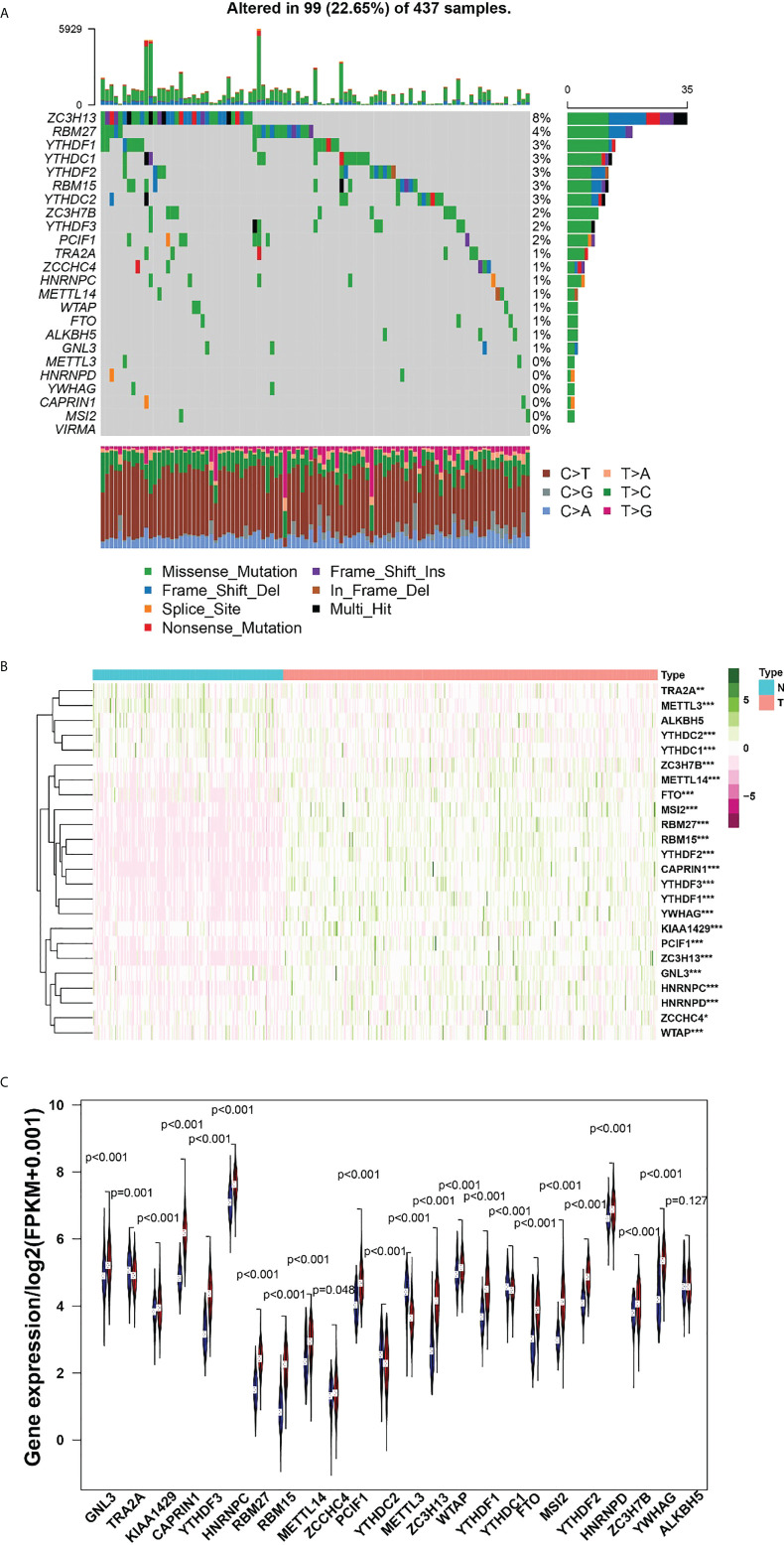
**(A)**, The mutation frequency in each regulator. **(B)** Heatmap of m6A RNA methylation regulator expression level in each sample. **(C)** The expression difference of m6A RNA methylation regulator between tumor and normal sample. *p<0.05; **p<0.01; ***p<0.001.

Among the 437 samples, a total of 99 samples were mutated, accounting for about 22.65%. Among them, ZC3H13 has the highest mutation rate, with a mutation rate of 8%. A total of 18 regulators were present in the sample with a mutation frequency greater than 1% (**Figure 1A**). Heatmap analysis of 437 samples revealed that m6A regulators were significantly elevated in gastric cancer tissues (**Figure 1B**). The expression difference of multiple m6A regulators, including GNL3, TRA2A, KIAA1429, CAPRIN1, YTHDF3, YTHDC2, METTL3, YYHDC1, YWHAG, HNRNPC, RBM27, RBM15, METTL14, ZCCHC4, PCIF1, ZC3H13, WTAP, YTHDF1, FTO, MSI2, YTHDF2, HNRNPD and ZC3H7B, was statistical significance between tumor samples and normal samples (**Figure 1C**).

### Correlations among PD-L1, PD-1, and m6A RNA methylation regulators

**Figure 2:**
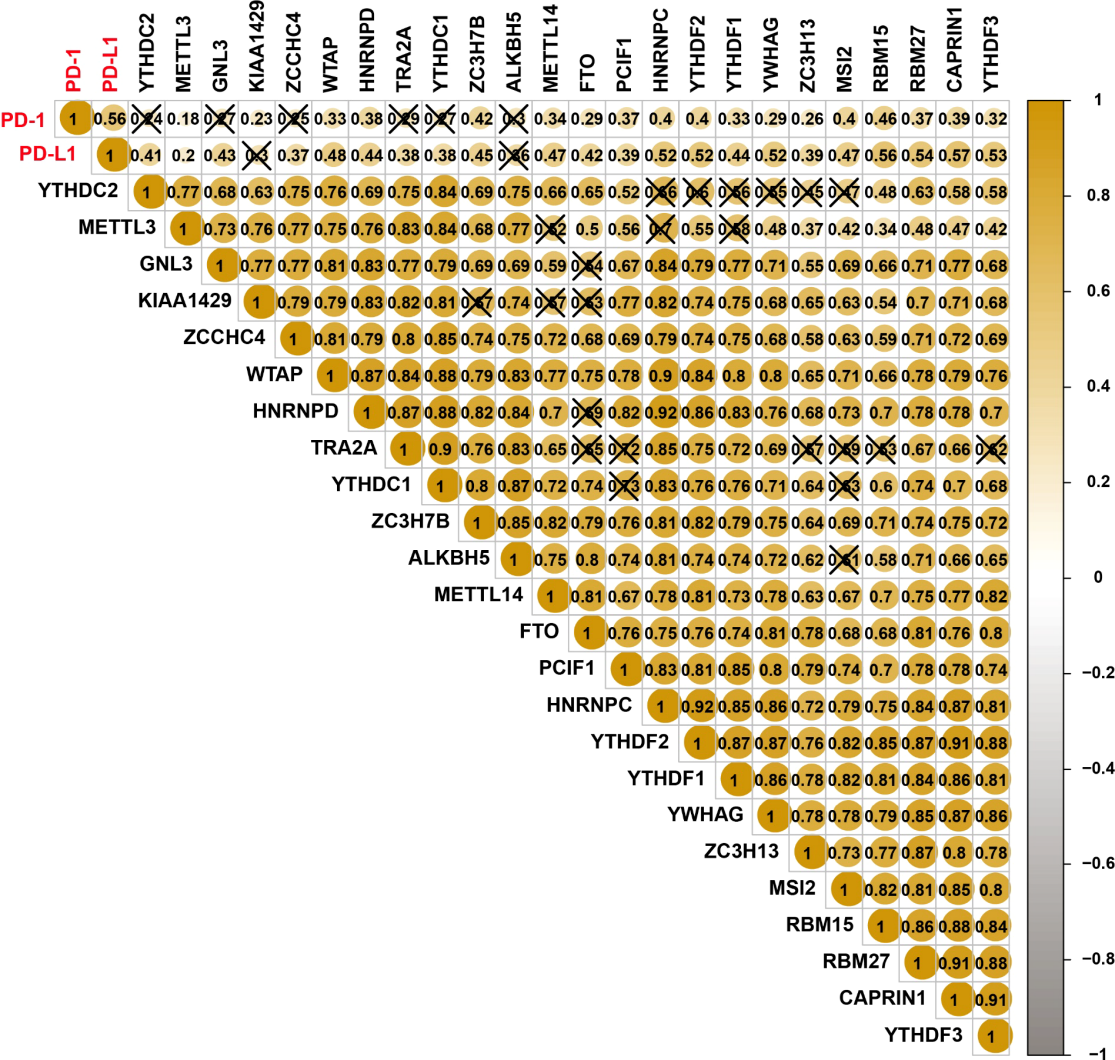
Correlations among PD-L1, PD1, and m6A RNA methylation regulators.

Spearman correlation was used to analyze the association of PD-1/PD-L1 and m6A regulators. The relationship between PD-1 and RBM15 was the strongest, and the correlation coefficient was 0.46 (**Figure 2**). The correlation between PD-1 and METTL3 was the weakest, with a correlation coefficient of 0.18. Similarly, the association between PD-L1 and CAPRIN1 was the strongest with a correlation coefficient of 0.57. However, the association between PD-L1 and METTL3 was the weakest with a correlation coefficient of 0.2 (**Figure 2**).

### Consensus clustering for m6A RNA methylation regulators with the characteristics and survival of patients with STAD

**Figure 3:**
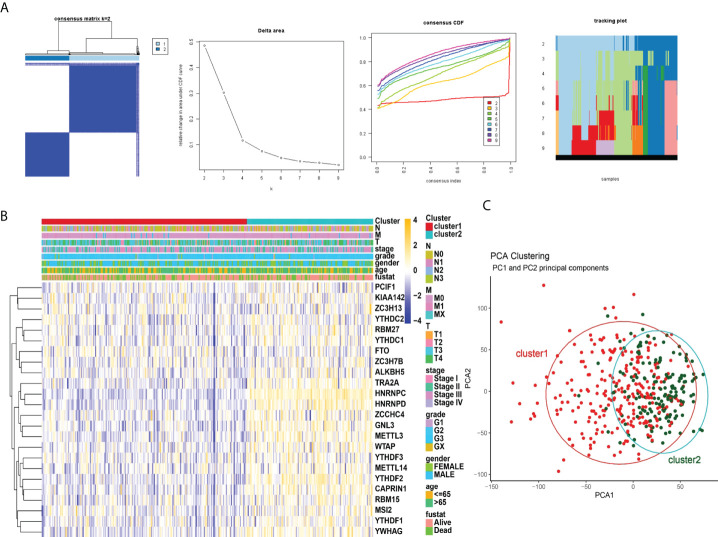
Correlation of consensus clustering for m6A RNA methylation regulators with the characteristics and survival of patients with STAD. **(A)**, Consensus clustering matrix for K=2; consensus clustering cumulative distribution function (CDF) for K=2 to 9; relative change in area under CDF curve for K=2 to 9. **(B)** Heatmap of correlation of m6A RNA methylation regulators with characteristics of STAD patients. **(C)**, PCA analysis of cluster1/2.

When K=2, the overlap between the two types is the least and the CDF value is the lowest (**Figure 3A**). Therefore, we divided the cohort into two clusters: cluster1 and cluster2. Subsequently, we analyzed the correlation of m6A RNA methylation regulators with the characteristics of gastric cancer patients, including TNM, stages, grade, gender, age (**Figure 3B**). Moreover, PCA analysis of cluster1 and cluster2 was illustrated (**Figure 3C**). In the following study, we will analyze the immune cells and m6A in two clusters.

### Infiltration level of immune cells and TME

**Figure 4:**
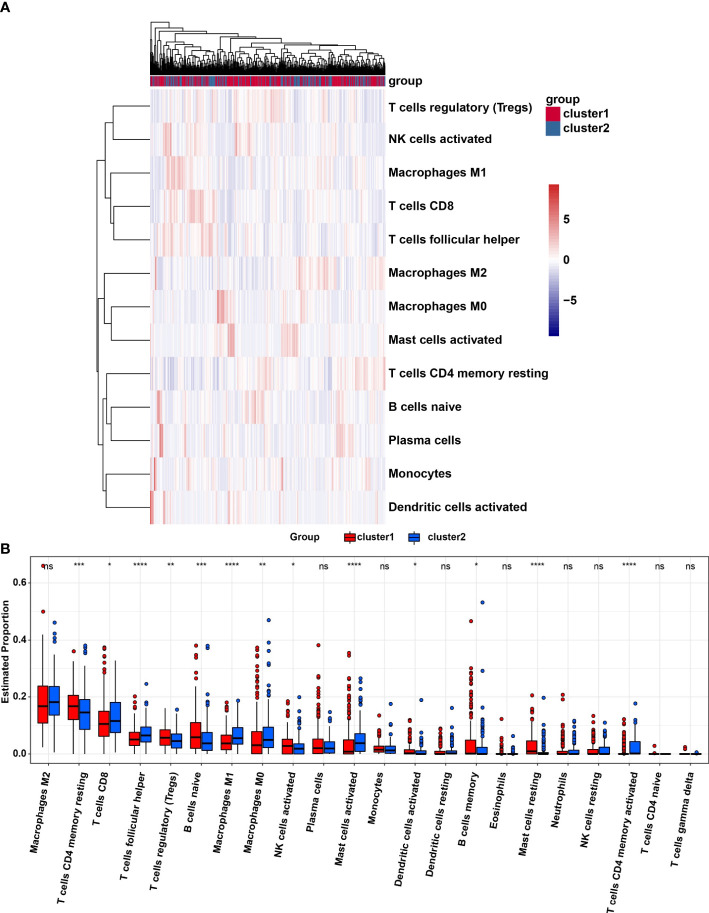
**(A)**, Heatmap of infiltrating levels of various immune cells in cluster1/2 in STAD. **(B)** Estimated proportion of 22 immune cell types in cluster1/2 in STAD. Ns, no significance; *p<0.05; **p<0.01; ***p<0.001; ****p<0.0001.

**Figure 5:**
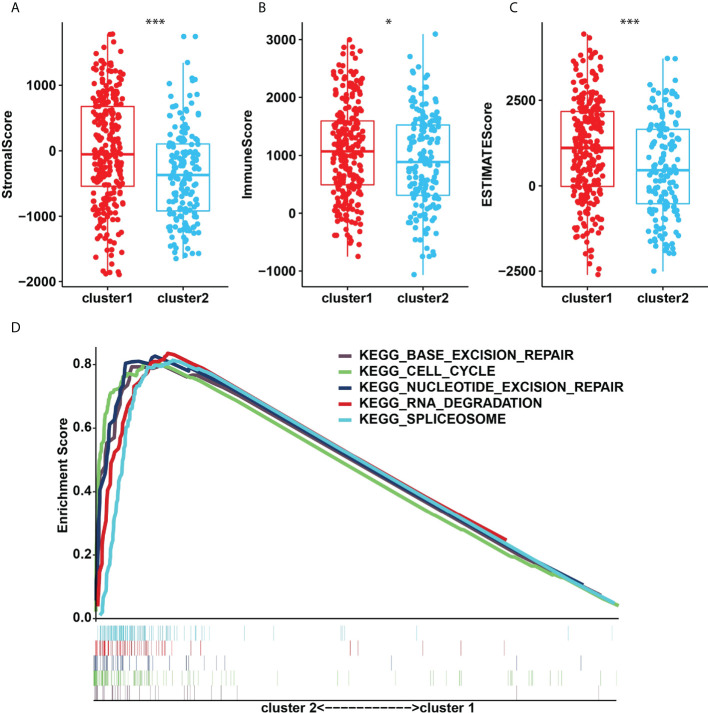
**(A)**, StromalScore. **(B)**, ImmunoScore. **(C)**, ESTIMATEScore in the cluster1/2 subtypes. **(D)**, The signaling pathways involved in cluster1 and cluster2. *p<0.05; ***p<0.001.

The TME was further analyzed to explore and calculate the infiltration of different immune cells in the gastric cancer samples. The infiltration level of cluster1 and cluster2 are shown in **Figure 4A**. Compared with cluster2, the estimated proportion of T cells CD4 memory resting, T cells regulatory, B cells naïve, NK cells activated, B cells memory, Dendritic cells activated, Mast cells resting in cluster1 was higher (**Figure 4B**). However, T cells CD8, T cells follicular helper, Macrophages M1, Macrophages M0, mast cells activated, T cells CD4 memory activated are lower in cluster1 (**Figure 4B**). To estimate the ratio of immune matrix components in the TME for each sample, we used the R package to estimate three scores in the form of the ESTIMATE algorithm: ImmuneScore, StromalScore and ESTIMATEScore. They were positively correlated with immunity, stroma, and the sum of the two, respectively. This means that the higher the corresponding score, the greater the ratio of the corresponding component in the TME (22). The results of **Figure 5** have shown that the StromalScore (**Figure 5A**), ImmuneScore (**Figure 5B**) and EstimateScore (**Figure 5C**) of cluster1 were all higher than those of cluster2, and the results were statistically significant. Clusters 2 were involved in five potential pathways, including base excision repair, cell cycle, nucleotide excision repair, RNA degradation, and spliceosome pathways (**Figure 5D**).

### Prognosis prediction of gastric cancer

**Figure 6:**
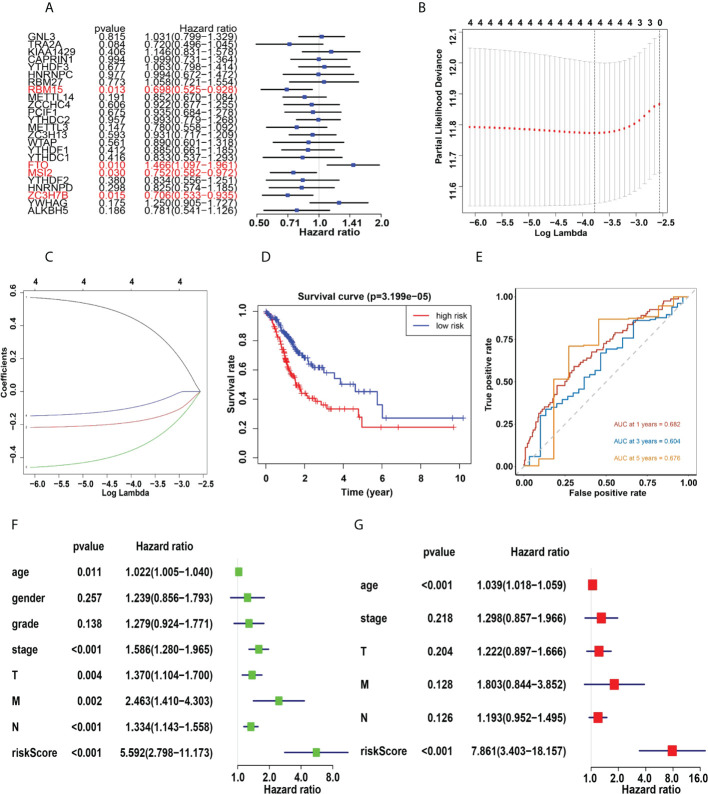
**(A)**, Univariate analysis of 24 regulators. **(B, C)** LASSO Cox regression algorithm. **(D)**, The Kaplan-Meier curve of high risk and low risk group. **(E)**, Time-dependent ROC curves. **(F, G)**, Univariate **(F)** and multivariate **(G)** Cox regression analysis of the RiskScores in TCGA.

**Figure 7:**
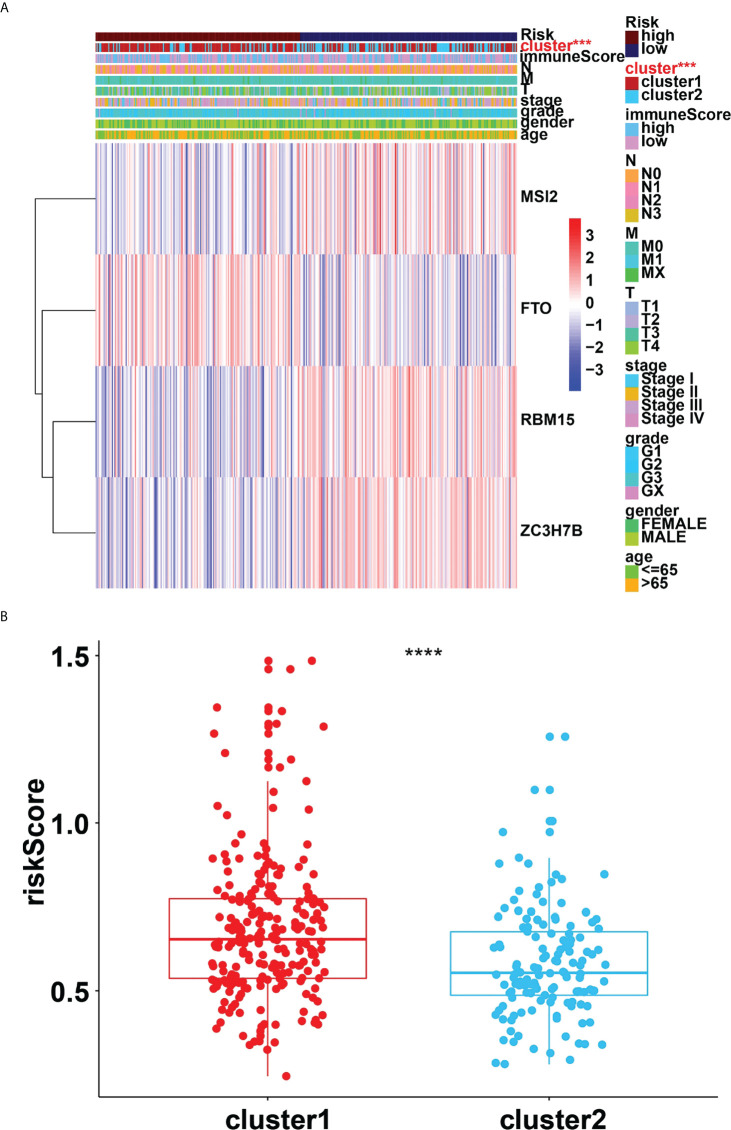
**(A)**, Heatmap of clinicopathologic features of STAD cohort. **(B)**, Distribution of RiskScores stratified by cluster1/2. ***p<0.001; ****p<0.0001.

Four genes, RBM15, FTO, MSI2 and ZC3H7B were identified as influencing the prognosis of gastric cancer patients in univariate analysis of 24 regulators (**Figure 6A**). To better understand the prognostic role of m6A RNA methylation regulators in gastric cancer, we performed Lasso Cox regression analysis on expression levels in the TCGA dataset (**Figures 6B, C**). The results showed that RBM15 (HR = 0.69, 95% CI = 0.52–0.92), FTO (HR = 1.46, 95% CI = 1.09–1.96), MSI2 (HR = 0.75, 95% CI = 0.58–0.97) and ZC3H7B (HR = 0.70, 95% CI = 0.53–0.93) was associated with prognosis in gastric cancer patients and the RiskSscore model was established. The formula was shown below: RiskScore =0.69* RBM15, +1.46*FTO, +0.75*MSI2, +0.70*ZC3H7B. Subsequently, the cohorts were divided into high-risk and low-risk groups based on RiskScores. Therefore, this study further identified the value of m6Ascore in predicting patient outcomes. Patients with low m6Ascore had a better survival rate (**Figure 6D**). The sensitivity and specificity of the prognostic model were tested by the AUC curve. The AUC at 1 years, 3 years and 5 years were 0.682, 0.604, 0.676, respectively (**Figure 6E**). Furthermore, RiskScore, stage status, T status, M status, and N status were all linked with OS in both univariate and multivariate Cox regression models. The probability of death increased when the RiskScore, stage status, T status, M status, and N status increased by univariate Cox regression (**Figure 6F**). The result of multivariate Cox regression analysis showed that the hazard ratio increased in patients with a high m6Ascore which indicated that the RiskScore was an independent powerful prognostic factor for the prognosis of OS in STAD (**Figure 6G**). The clinicopathologic features of the STAD cohort have been displayed in **Figure 7A**. The RiskScore of cluster1 is higher than that of cluster2 (**Figure 7B**). There were significant differences in m6Ascore between gene clusters. The RiskScore for gene cluster1 is high, while the score for gene cluster2 is low.

### Expression of PD-1 and PD-L1

**Figure 8:**
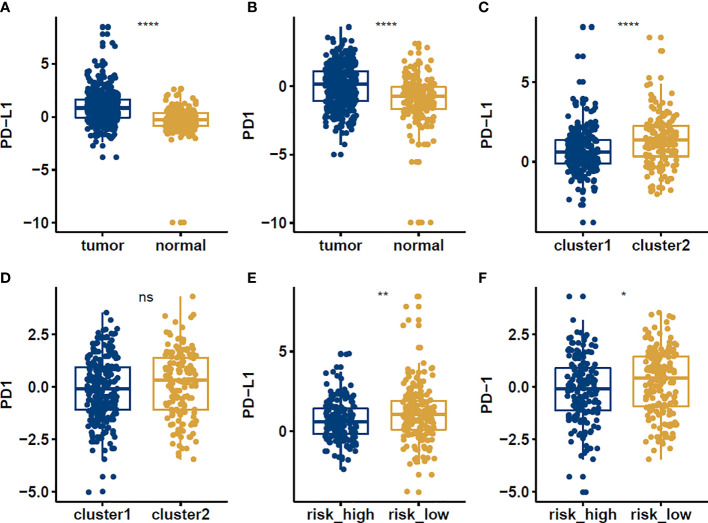
The expression levels of PD-1 and PD-L1 in normal vs tumor **(A, B)**, cluster1 vs cluster2 **(C, D)**, high risk vs low risk **(E, F)** groups in STAD patients. *p<0.05; **p<0.01; ****p<0.0001, ns: no significance.

**Figure 9:**
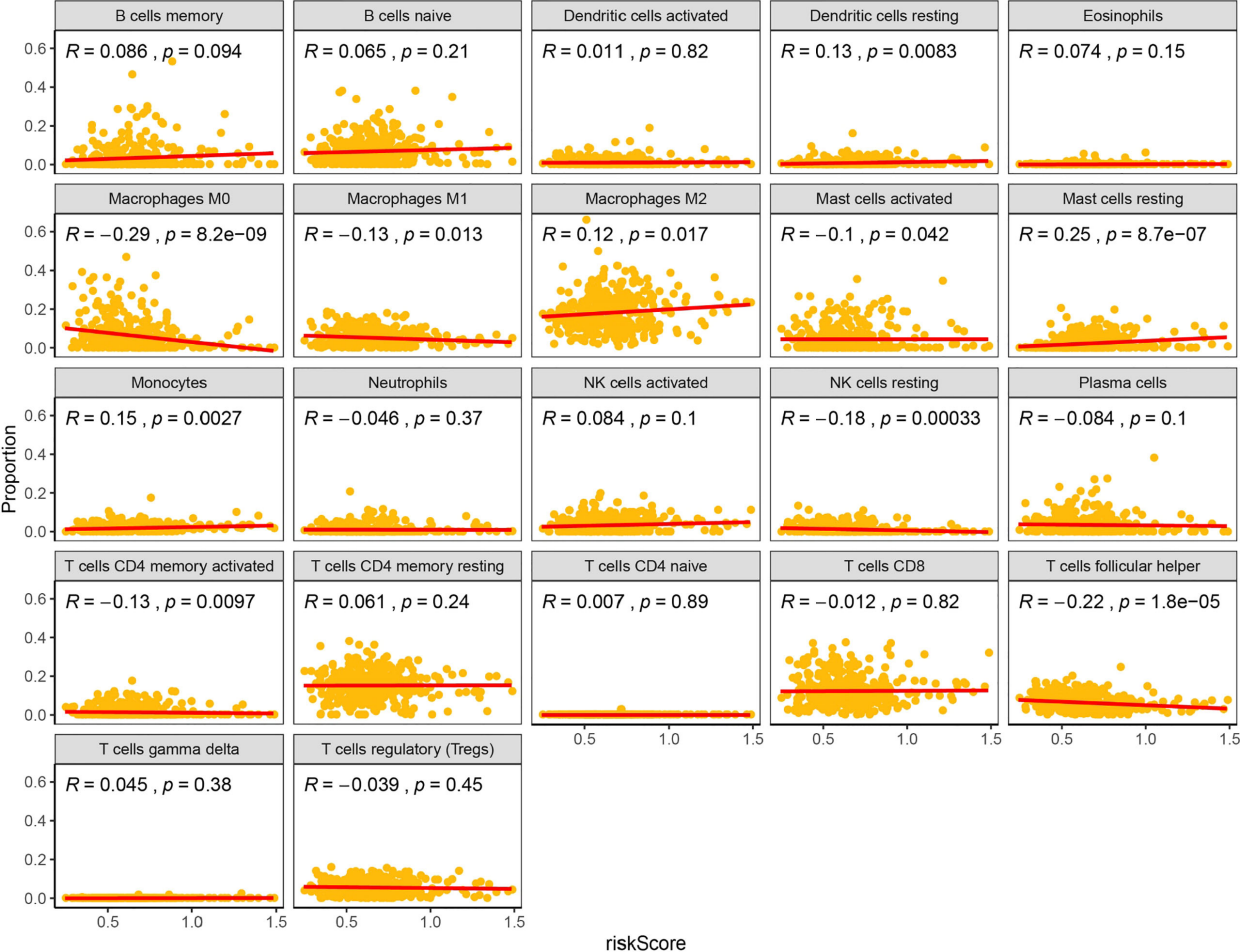
Relationships between the RiskScore and infiltration abundances of 22 immune cell types.

PD-1 and PD-L1 were highly expressed in tumor cells (**Figures 8A, B**). Meanwhile, the results indicated that the expression levels of PD-L1 were higher in cluster1 compared to cluster2 (**Figures 8C, D**). PD-1 and PD-L1 were highly expressed in the low-risk group (**Figures 8E, F**). Patients with low m6Ascore showed significantly higher expression of PD-L1, indicating a potential response to immunotherapy against PD-1/PD-L1. Moreover, the relationships between the RiskScore and infiltration abundances of 22 immune cell types were analyzed. Dendritic cells resting, Macrophages M0, Macrophages M1, Macrophages M2, Mast cells activated, Mast cells resting, Monocytes, NK cells resting, T cells CD4 memory activated, T cells follicular helper were associated with the RiskScore in gastric cancer patients (**Figure 9**).

### METTL3 regulates the expression of PD-L1 in gastric cancer cells

**Figure 10:**
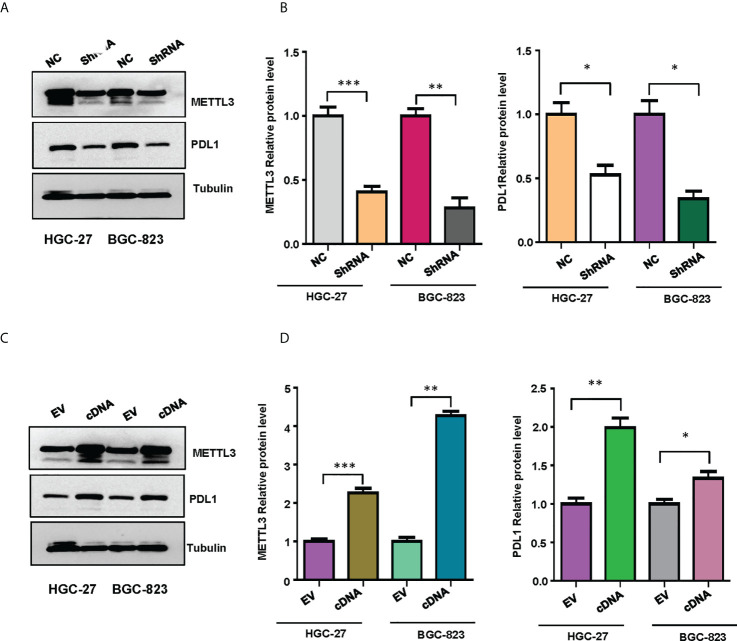
The expression of METTL3 and PD-L1 in gastric cancer cells was illustrated. **(A)**, Western blotting was performed to measure the expression of METTL3 and PD-L1 in HGC-27 and BGC-823 cells after METTL3 downregulation. **(B)**, Quantitative data were shown for panel **(A)**. **(C)**, Western blotting was performed to measure the expression of METTL3 and PD-L1 in HGC-27 and BGC-823 cells after METTL3 overexpression. **(D)**, Quantitative data were shown for panel **(C)**. *p<0.05; **p<0.01; ***p<0.001.

To validate the relationship between METTL3 and PD-L1 in gastric cancer cells, we performed the western blotting to measure the expression levels of PD-L1 in HGC-27 and BGC-823 cells after METTL3 overexpression or depletion. We found that downregulation of METTL3 reduced the expression of PD-L1 in both HGC-27 and BGC-823 cells (**Figures 10A, B**). Moreover, overexpression of METTL3 increased the expression levels of PD-L1 in gastric cancer cells (**Figures 10C, D**). Our finding suggests that METTL3 could regulate the expression of PD-L1 in gastric cancer cells.

## Discussion

Gastric cancer is the second-leading cause of cancer death in the world. Endoscopic therapy, surgery, chemotherapy, and radiotherapy are currently the most common treatments for gastric cancer (23, 24). However, the efficacy of chemotherapy and targeted therapy in patients with gastric cancer is limited, and the prognosis is poor (25). Besides, the recurrence and metastasis rate is high, and the 5-year survival rate is 15% - 25% (26, 27). The median time to death ranged from 9 to 23 months (28). Although advances in the treatment of advanced gastric cancer have given hope, there are still many unanswered questions in the field of advanced gastric cancer immunotherapy (29). As a result, additional investigation will be required.

The monitoring and regulation of the TME in gastric cancer is an effective way to prevent and treat the disease. At this time, the knowledge of the microenvironment of gastric cancer is insufficient, and it cannot accurately reflect changes in the whole body (30). Although m6A methylation has been associated with the initiation and progression of a variety of cancers, there have been few investigations on gastric cancer (31). The major goal of this study is to explore the connection between m6A RNA methylation regulators, PD-L1, prognosis, and TIME in gastric cancer. Through this study, we found that the majority of m6A regulatory factors are considerably overexpressed in gastric cancer tissues. Besides, the study has demonstrated two gastric cancer subgroups (Cluster1/2) based on consensus clustering of 21 m6A regulators. PD-L1 and PD-1 expression levels were significantly higher in gastric cancer tissues, and they were significantly linked with METTL3, WTAP, HNRNPD, ZC3H7B, METTL14, FTO, PCIF1, HNRNPC, YTHDF1 and YTDHF2. WTAP enhanced the Warburg effect *via* regulation of HK2 stability in gastric cancer (32). Overexpression of WTAP resulted in poor survival of gastric cancer patients *via* modulation of tumor-associated lymphocyte infiltration (33). FTO accelerated cell growth and metastasis *via* regulation of caveolin-1 and ITGB1 and metabolic regulation of mitochondrial dynamics in gastric cancer (34, 35). FTO could be a helpful therapeutic target for gastric cancer patients (36). PCIF1 was involved in aggressiveness of gastric cancer cells *via* suppression of TM9SF1 mRNA translation (37). HNRNPC has been identified as a chemoresistance biomarker for gastric cancer patients (38). In addition, depletion of YTHDF1 elevated sensitivity to antitumor immunity *via* recruitment of mature dendritic cells in gastric tumors (39). YTHDF2 reduced cell growth by targeting FOXC2 pathway in gastric cancer (40). It is still unclear whether these m6A regulators are associated with PD1 and PDL1 expressions in gastric cancer.

Cluster1 has a large increase in CD4 memory resting T cells, regulatory T cells, naïve B cells, active NK cells, and resting Mast cells. Cluster1 and Cluster2 were proved to be involved in numerous critical signaling pathways, including base excision repair, cell cycle, nucleotide excision repair, RNA degradation, and spliceosome pathways. RBM15, FTO, MSI2, and ZC3H7B were identified as risk signatures using Cox regression analysis and LASSO analysis. Gastric cancer RiskScores based on prognostic factors have been found as independent prognostic indicators. The amount of tumor-infiltrating immune cells is dynamically affected by changes in the copy number of m6A methylation regulators associated with TIME.

Through our study, we found that TRA2A, METTL3, ALKBH5 and TYHDC2 were significantly down-regulated in gastric cancers cells. Studies have discovered that METTL3 is highly expressed in gastric cancer cells, and that knockdown of METTL3 dramatically reduced cell proliferation, migration, and invasion in human gastric and HCC cancer cells (41, 42). METTL3 induced proliferation, migration, angiogenesis and tumorigenesis *via* inhibition of ADAMTS9 and modification of YAP1 and KLF2 in gastric cancer (43–45). METTL3 facilitated oxaliplatin resistance *via* enhancing the stability of PARP1 mRNA in gastric cancer stem cells (46). METTL3 was also taken part in linc00240-induced gastric cancer progression *via* targeting miR-338-5p (47). Upregulation of METTL3 can inhibit renal cell carcinoma proliferation, migration and cell cycle (48). These inconsistent studies suggest that METTL3 may play opposite roles in different tumor cells. Possibly, this may be due to the complexity tumor microenvironment and tumor heterogeneity (49). Besides, METTL14 was down-regulated in gastric cancer tissue samples, and its low expression could be a prognostic factor for poor survival in gastric cancer patients (50). Ectopic expression of METTL14 significantly inhibited gastric cancer cell growth and invasion *in vitro* and *in vivo*, while downregulation of METTL14 had the opposite effect (50). Subsequently, in order to further analyze the relationship between PD-1 and m6A regulators in gastric cancer, we found that in gastric cancer cells, PD-1/L1 and METTL3, WTAP, HNRNPD, ZC3H7B, METTL14, FTO, PCIF1, HNRNPC, YTHDF2, YTHDF1, YWHAG, ZC3H13 and MSI2A were significantly correlated in gastric cancer cells. One recent study demonstrated that low m6Ascore was associated with enhanced response to anti-PD-1/L1 immunotherapy, suggesting that cohort with a lower m6Ascore has a more pronounced clinical effect (51).

Noncoding RNAs have been reported to participate in cancer development *via* regulating TME in various types of cancers, including gastric cancer (52–55). Another study indicated that m6A-related lncRNAs regulate TIME infiltration and govern immunotherapy and chemotherapy for gastric cancer patients (56). Moreover, risk signatures are associated with a variety of tumor-infiltrating immune cells (56). Studies have also proved that PD-1 and CTLA4 have highly expressed in the low-risk group, while PD-L1, LAG3 and IDO1 had no distinct expression differences in the low-risk group (56). In our study, PD-1 and PD-L1 were more expressed in the low-risk group. Patients with low m6Ascore showed significantly higher expression of PD-L1, indicating a potential response to immunotherapy against PD-1/L1.

The biological characteristics of the metabolic environment of gastric cancer are very beneficial to the proliferation and invasion of tumors. It is an important factor that promotes the occurrence, transformation and metastasis of malignant tumors. These features include the following aspects: hypoxic microenvironment, acidosis microenvironment, interstitial hypertension, immune-inflammatory response (18, 57, 58). Gastric cancer cells can regulate the expression of some transcription factors and tumor-related genes in the microenvironment to affect the biological characteristics of tumor cells and adapt to the hypoxia environment. HIF-1 is one of the important transcription factors in the tumor hypoxic microenvironment (59). In our research, CD4 memory resting T cells was highly expressed in cluster1 and CD8+ T cell was highly expressed in cluster2. A study confirmed that suppressing gastric cancer by increasing the volume of CD8+ T cells may be critical. Other recent study has found that the number of CD4+ T cell fractions is increased in gastric cancer, while the CD8+ T cell fraction is decreased. Tumor antigen-specific CD8+ T cells are negatively regulated by PD-1 in gastric cancer (60).

Several studies have revealed that METTL3 regulated the expression of PD-L1 in bladder cancer and breast cancer (61, 62). Here, our study reported that METTL3 governed the expression of PD-L1 in gastric cancer cells. Lastly, this study has numerous advantages. It is the first to use bioinformatics methods to describe the link between m6A PD-1 and the tumor microenvironment of gastric cancer. In addition, this work developed the m6A model for stomach cancer prognosis, which has substantial therapeutic implications. However, this study has several disadvantages and limitations. The clinical sample size for this study was insufficient because it solely used TCGA stomach cancer data. More data and high-quality samples will be required in future studies. The evidence comes mostly from the TCGA cohort and will need to be confirmed in cell culture system and in mouse model and clinical samples. For instance, our bioinformatic data indicated the weak association between PD-L1 and METTL3. However, in line with other studies, we found that METTL3 regulated the expression of PD-L1 in gastric cancer. Finally, other m6A regulators may exist, and the interaction between other variables, PD-1, and the tumor microenvironment need to be investigated further.

## Conclusions

Gastric cancer RiskScores based on prognostic factors have been found as independent prognostic indicators. Tumor-infiltrating immune cells are dynamically affected by changes in the copy number of m6A methylation regulators associated with TIME.

## Data availability statement

The original data presented in the study are included in the article and **Supplementary Material**. Further inquiries can be required from the corresponding authors.

## Author contributions

ZX, QC performed the experiments, analyzed the data and wrote the manuscript. LS, CZ, WL analyzed the data and reviewed the study. ZX and PW designed the study. PW edited the manuscript and supervised this study. All authors edited and approved the final manuscript.

## Funding

This work was supported by Key Laboratory of Prevention, Diagnosis and Therapy of Upper Gastrointestinal Cancer of Zhejiang Province (2022E10021) and the National Natural Science Foundation of China (81973634).

## Conflict of interest

The authors declare that the research was conducted in the absence of any commercial or financial relationships that could be construed as a potential conflict of interest.

## Publisher’s note

All claims expressed in this article are solely those of the authors and do not necessarily represent those of their affiliated organizations, or those of the publisher, the editors and the reviewers. Any product that may be evaluated in this article, or claim that may be made by its manufacturer, is not guaranteed or endorsed by the publisher.
