# Supplementary figures and images for: Expression profiles of m6A RNA methylation regulators, PD-L1 and immune infiltrates in gastric cancer
Source: Front Oncol. 2022 Aug 8;12:970367. doi: 10.3389/fonc.2022.970367 (PMC9393729; doi:10.3389/fonc.2022.970367)

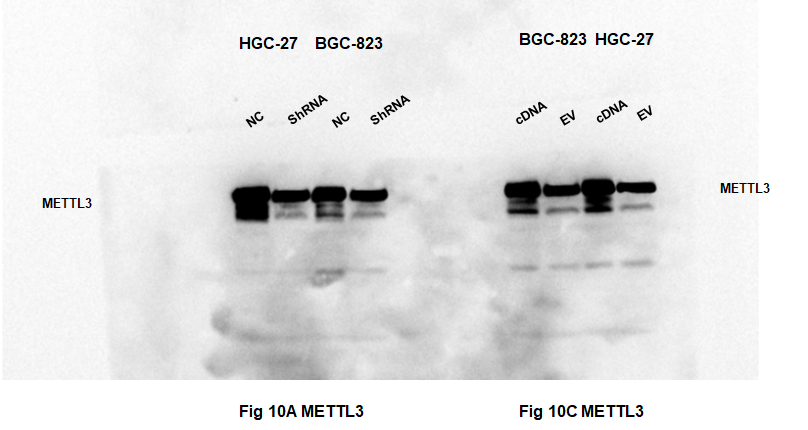

Supplement: Supplementary file 1 [file Image_1.tif]

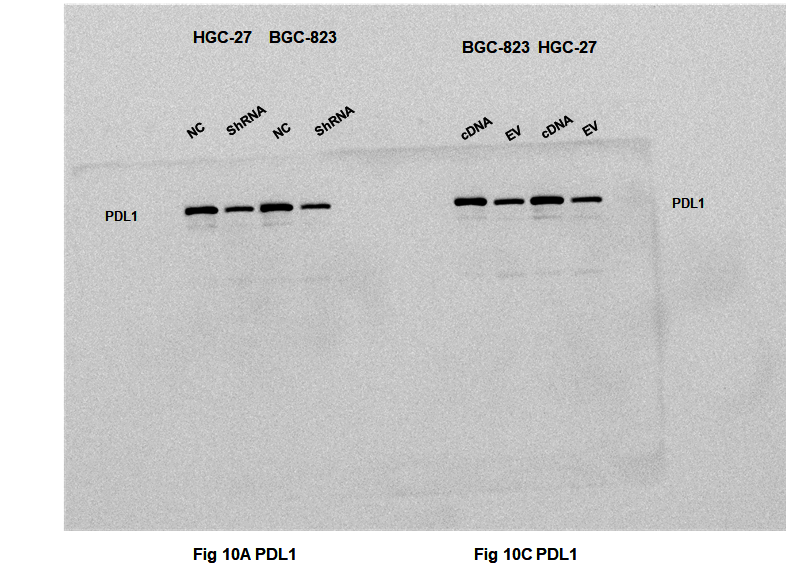

Supplement: Supplementary file 2 [file Image_2.tif]

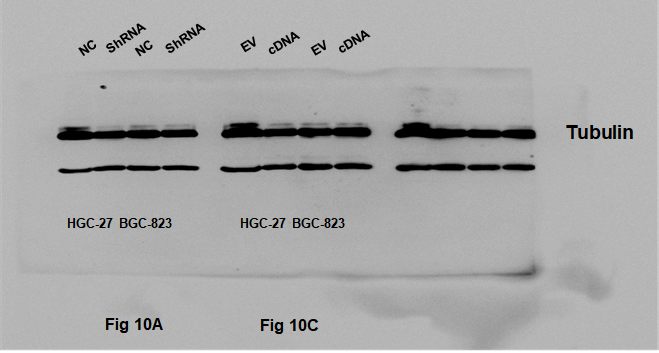

Supplement: Supplementary file 3 [file Image_3.tif]
